# Safety, tolerability and pharmacokinetics of forsythin in healthy subjects: a double-blinded, placebo-controlled single-dose and multiple-dose escalation and food effect study

**DOI:** 10.1080/07853890.2023.2274512

**Published:** 2023-11-19

**Authors:** Cuiyun Li, Min Wu, Hong Zhang, Xiaoxue Zhu, Li Fu, Shuo Wang, Mingming Lu, Dafang Zhong, Yanhua Ding

**Affiliations:** a Phase I Clinical Trial Unit, First Hospital, Jilin University, Changchun, China; b Dalian Fusheng Institute of Natural Medicine, Dalian, China; c State Key Laboratory of Drug Research, Shanghai Institute of Materia Medica, Chinese Academy of Sciences, Shanghai, China

**Keywords:** Forsythin, pharmacokinetics, safety, tolerability, food effect

## Abstract

**Background:**

Forsythin, an active compound from Forsythiae Fructus, has the potential to treat the common cold and influenza through its antipyretic-analgesic, anti-inflammatory and antiviral effects. The safety, tolerability and pharmacokinetic (PK) profile of forsythin were evaluated in healthy Chinese subjects.

**Methods:**

This phase 1a study included three parts: double-blind, randomized, placebo-controlled single-ascending-dose (SAD) (50, 100, 200, 400, 600 or 800 mg), food effect investigation (100 mg) and multiple-ascending-dose (MAD) (50, 100 or 200 mg TID for 5 days).

**Results:**

Forsythin is safe and tolerable in healthy Chinese subjects. The rates of adverse events (AEs) in the forsythin cohort were similar to those in the placebo cohort. Forsythin is well-absorbed after single or multiple doses and is extensively metabolized. The primary metabolites were aglycone M1, M1 sulphate (M2) and M1 glucuronide (M7). Exposure to forsythin (100 mg) was higher after food intake by approximately 1.4-fold, whereas M2 and M7 did not change. The steady state was reached around three days in the MAD study. Forsythin, M2 and M7 accumulation on day 5 was 1, 3 and 2, respectively.

**Conclusions:**

The safety and PK profiles of forsythin support further evaluation of its efficacy in individuals with the common cold or influenza.

## 1. Introduction

Influenza, a highly contagious disease, is a global health, medical and economic health problem. Worldwide, the annual number of deaths resulting from influenza is approximately 290,000–650,000 [1]. The symptoms can range from mild to severe with high fever, chills, muscle pain, pneumonia and even death [2].

Forsythiae Fructus (FF, Lianqiao in Chinese) is widely used for the clinical treatment of respiratory infectious diseases in China, Japan and Korea [3]. FF is used as an active ingredient in more than 100 Chinese herbal preparations [4]. Forsythin, an active compound from FF, belongs to a class of natural glycosidic lignan compounds [5].

Forsythin has several pharmacological effects, including antiviral, antipyretic, anti-fibrotic [6] and anti-inflammatory effects on osteolysis, acute kidney injury, lung injury and traumatic brain injury in mice [7–10]. Moreover, forsythin has been shown to attenuate lipid accumulation and lower the weight of obese mice in previous studies [11,12].

Forsythin is being developed as a novel drug to treat the common cold and influenza. In preclinical studies, forsythin has shown antiviral effects, antipyretic–analgesic and anti-inflammatory effects. The antipyretic–analgesic effect results from sweating, antiviral effects and decreased levels of prostaglandin E2 (PGE2), Interleukin-1β (IL-1β) and cyclic adenosine monophosphate (cAMP) in the brain tissue of model animals. The antiviral effect was related to the inhibition of viral proliferation and reduction of inflammatory cytokines. Forsythin showed dose-dependent antiviral effects in mouse models. Based on preclinical pharmacodynamic results, two metabolites, aglycone M1 and M1 sulphate (M2), were found to be active. The activity of M2 was higher than that of M1 but lower than that of forsythin. The no-observed-adverse-effect level of forsythin was 6.4 g/kg for Sprague Dawley rats and 0.8 g/kg for Beagle dogs. In different animal models, the effective dose was 6.75–27 mg/kg of forsythin. Forsythin showed good antiviral effects at a dose range of 16–26 mg/kg. Therefore, the human equivalent effective dose is 65–260 mg. Preclinical data of forsythin indicated that the maximum tolerable starting dose in initial clinical trials in healthy adult volunteers was 2592 mg, with a 10-fold tolerability factor [13].

The objective of this phase 1a trial was to assess the safety, tolerability and pharmacokinetic (PK) profiles as well as the food effect of forsythin. This study helped select the dosages for the phase 2 study, which is currently conducted in China.

## 2. Results

### 2.1. Volunteers

A total of 112 healthy Chinese subjects out of 366 screened subjects were eligible for this study and completed the safety analysis after completion of the study. All the volunteers completed the study. The demographics of the volunteers, including age, weight and body mass index (BMI), are summarized in Table 1.

**Table 1.** Demographics of the volunteers in this study.

Cohort	Age (years)	Gender		Height (cm)	Weight (kg)	Body-mass index (kg/m^2^)
		Male	Female			
*Single-ascending-dose*						
Placebo (*n* = 12)	32.9 (8.6)	6 (50.0)	6 (50.0)	163.4 (7.6)	57.3 (6.5)	21.5 (2.1)
50 mg (*n* = 10)	34.3 (7.0)	5 (50.0)	5 (50.0)	162.7 (7.2)	56.1 (5.4)	21.2 (1.0)
100 mg (*n* = 8)	34.1 (6.2)	4 (50.0)	4 (50.0)	160.9 (8.8)	58.7 (7.2)	22.7 (1.8)
200 mg (*n* = 8)	31.0 (8.2)	4 (50.0)	4 (50.0)	164.9 (6.8)	62.1 (8.7)	22.8 (2.3)
400 mg(*n* = 8)	26.9 (5.1)	4 (50.0)	4 (50.0)	164.5 (10.7)	57.6 (11.2)	21.1 (1.9)
600 mg (*n* = 8)	29.9 (8.4)	4 (50.0)	4 (50.0)	163.4 (8.8)	59.0 (7.0)	22.1 (1.6)
800 mg (*n* = 8)	32.2 (7.7)	4 (50.0)	4 (50.0)	163.9 (9.1)	62.6 (11.1)	23.1 (2.0)
*Multiple-ascending-dose*						
Placebo (*n* = 9)	38.0 (5.7)	5 (55.6%)	4 (44.4%)	163.0 (6.5)	59.9 (6.0)	22.8 (1.7)
50 mg (*n* = 9)	33.1 (7.4)	5 (55.6%)	4 (44.4%)	164.9 (8.1)	60.4 (8.5)	22.1 (1.6)
100 mg (*n* = 9)	33.7 (8.7)	4 (44.4%)	5 (55.6%)	167.3 (9.4)	61.4 (8.4)	22.0 (2.7)
200 mg (*n* = 9)	31.1 (7.3)	4 (44.4%)	5 (55.6%)	160.8 (10.3)	59.8 (11.3)	23.0 (2.7)
*Food effect study*						
A cohort (*n* = 7)	26.0 (8.0)	3 (42.9%)	4 (57.1%)	164.6 (6.6)	58.6 (8.9)	21.6 (2.1)
B cohort (*n* = 7)	29.6 (5.9)	4 (57.1%)	3 (42.9%)	162.4 (11.7)	61.1 (6.9)	23.3 (3.2)

Data are *n* (%) or mean (SD).

### 2.2. Tolerability and safety

Forsythin was well tolerated by healthy Chinese subjects at each dose investigated in this study. No severe or serious adverse events (AEs) were observed in any subject. The safety results of the AEs are summarized in Table 2.

**Table 2.** Adverse events summary after administration of forsythin by dose cohort (*n* = 112).

	Single-ascending-dose							Multiple-ascending-dose				Food effect study	
Preferred term	50 mg	100 mg	200 mg	400 mg	600 mg	800 mg	Placebo	50 mg TID	100 mg TID	200 mg TID	Placebo	A cohort (*n* = 7)	B cohort (*n* = 7)
	(*N* = 10)	(*N* = 8)	(*N* = 8)	(*N* = 8)	(*N* = 8)	(*N* = 8)	(*N* = 12)	(*N* = 9)	(*N* = 9)	(*N* = 9)	(*N* = 9)		
	*n* (%)	*n* (%)	*n* (%)	*n* (%)	*n* (%)	*n* (%)	*n* (%)	*n* (%)	*n* (%)	*n* (%)	*n* (%)		
Over with TEAE	1 (10.0)	0	0	2 (25.0)	1 (12.5)	0	3 (25.0)	5 (55.6)	7 (77.8)	2 (22.2)	5 (55.6)	0	1 (7.1)
ALT increased	0	0	0	0	0	0	0	1 (11.1)	0	0	1 (11.1)	0	0
AST increased	0	0	0	0	0	0	0	0	1 (11.1)	0	0	0	0
CK increased	0	0	0	0	0	0	0	0	0	1 (11.1)	0	0	0
CK-MB increased	0	0	0	0	0	0	0	0	0	1 (11.1)	0	0	0
Serum bilirubin increased	0	0	0	0	0	0	0	0	1 (11.1)	0	1 (11.1)	0	1 (7.1)
Hypertriglyceridemia	0	0	0	0	0	1 (12.5)	0	0	1 (11.1)	0	0	0	0
Hypoalbuminemia	0	0	0	0	0	0	0	2 (22.2)	1 (11.1)	0	0	0	0
Hypokalemia	0	0	0	1 (12.5)	0	0	1 (8.3)	0	0	2 (22.2)	2 (22.2)	0	0
β-N-Acetyl-D-glucosami-nidase increased	0	0	0	0	0	0	0	1 (11.1)	2 (22.2)	0	0	0	0
Anemia	0	0	0	0	0	0	0	0	1 (11.1)	0	0	0	0
Urinary RBC positive	0	1 (12.5)	0	0	0	0	0	1 (11.1)	1 (11.1)	0	0	0	0
Proteinuria	0	0	0	0	0	0	1 (8.3)	1 (11.1)	0	0	0	0	0
Urinary tract infection	1(10.0)	0	0	1 (12.5)	0	0	1 (8.3)	1 (11.1)	2 (22.2)	0	1 (11.1)	0	0
Headache	0	0	0	0	1 (12.5)	0	0	0	0	0	0	0	0
Pharyngalgia	0	0	0	0	1 (12.5)	0	0	0	0	0	0	0	0
Dorsalgia	0	0	0	0	0	0	0	0	1 (11.1)	0	0	0	0
Epigastric pain	0	0	0	0	0	0	0	0	1 (11.1)	0	0	0	0
Supraventricular arrhythmia	0	0	0	0	0	0	0	1 (11.1)	0	0	0	0	0
Ventricular extrasystole	0	0	0	0	0	0	0	0	1 (11.1)	0	0	0	0

*N* = number of subjects analyzed; *n* = number of subjects.

In the single-ascending-dose (SAD) study, eight AEs were reported by seven subjects (11.3%), including five in the forsythin cohort (four subjects, 8.0%) and three in the placebo cohort (three subjects, 25.0%), of which one was determined to be adverse drug reaction (ADR). The only ADR observed in the 600 mg cohort. All AEs were classified as grade 1. All the subjects recovered within several days. In the food effect study, one subject reported an increase in serum bilirubin level of 2.

Overall, the incidence rates of AEs in the forsythin and placebo cohorts were 52.8% and 55.6%, respectively, in the multiple-ascending-dose (MAD) study. Most AEs were abnormal in clinical laboratory tests. One CK increase event reported by one subject on day 6 in the 100 mg MAD cohort was classified as grade 4. It was not considered to be related to the drug, as the subject performed strenuous exercise. The same subject also reported an increase in CK-MB levels (grade 2) and recovered without any intervention. Four (14.8%) and three (33.3%) subjects reported ADRs in the forsythin and placebo cohorts, respectively. The severity of all ADRs was not dose dependent.

No clinically significant changes were observed in vital signs and ECG.

### 2.3. PK of forsythin

#### 2.3.1. SAD and food effect study

Mean forsythin and metabolite plasma concentration–time profiles and PK parameters after a single dose of forsythin have been presented in a previous report [14]. Following a single administration of forsythin up to 800 mg, forsythin was absorbed rapidly. *C*_max_ and AUC_0–∞_ increased in the dose range of 50–800 mg. However, the estimated values of the power model exponent *β* for *C*_max_ and AUC_0–∞_ for forsythin were 0.34 and 0.36, respectively. Increasing the forsythin dose in the subjects resulted in a less than dose-proportional increase in exposure. The exponent *β* for the *C*_max_ and AUC_0–∞_ of M2 and M7 were approximately 1. The plasma concentration–time profiles of M1, M2 and M7 showed two peaks, indicating enterohepatic circulation.

**Figure 1.**
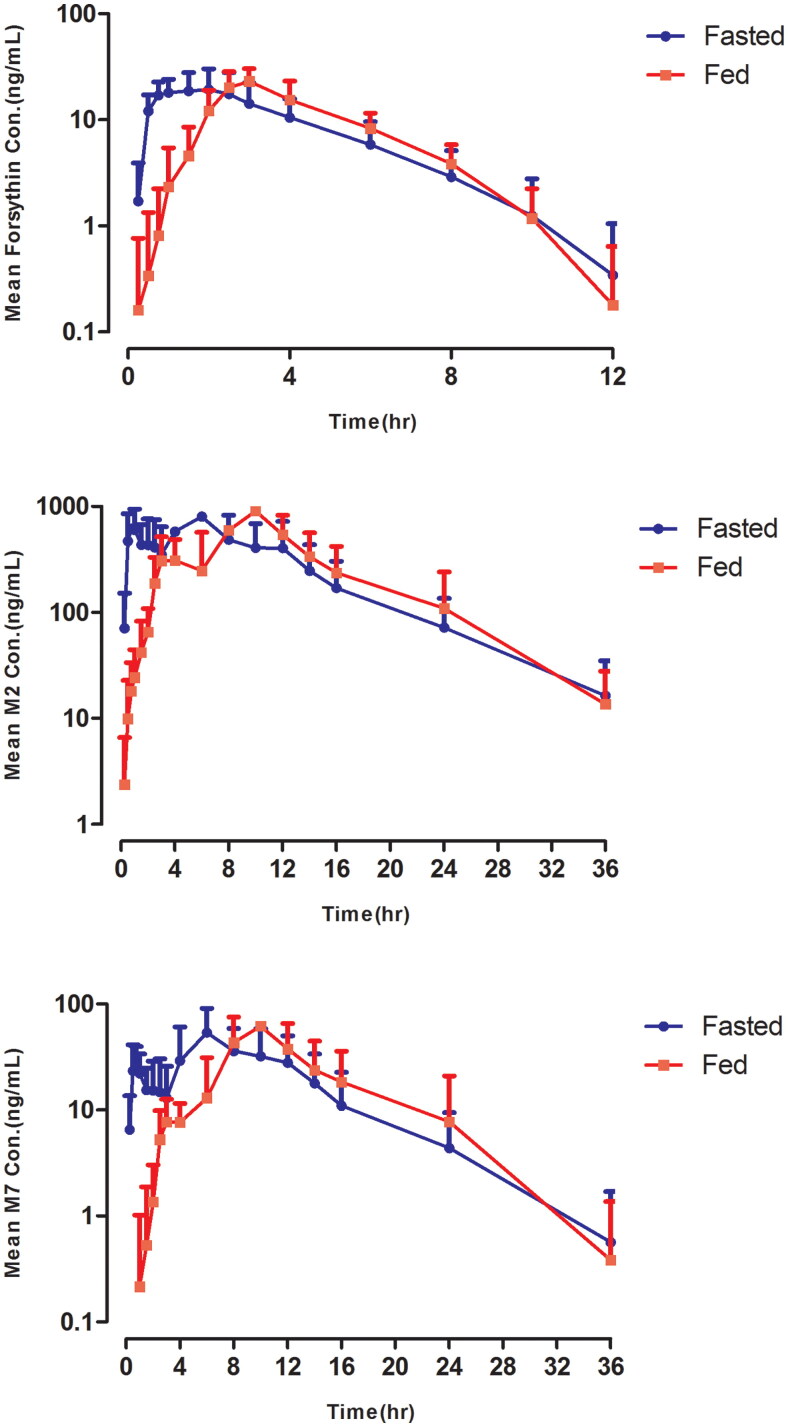
Pharmacokinetic profiles of forsythin, M2 and M7 in food effect study (100 mg).

The mean forsythin, M2 and M7 plasma concentration–time profiles in the food effect study are shown in Figure 1. The *T*_max_ were delayed by approximately 4 h. The geometric LSM ratios (90% CI) of *C*_max_ and AUC_0–∞_ for the analytes are listed in Table 3. Bioavailability was similar in the fed and fasted states, indicating that forsythin could be taken after meals.

**Table 3.** Geometric mean ratios for plasma pharmacokinetic parameters of forsythin under fed and fasted states in the food effect study.

Analyte	PK parameters	Geometric mean (Fed)	Geometric mean (Fast)	Geometric mean ratios (Fed/Fast)	CI_90_Lower	CI_90_Upper
Forsythin	AUC_0–∞_ (h·ng/mL)	117	85.3	136.90	119.65	156.63
	AUC_0–t_ (h·ng/mL)	112	79.6	141.06	121.55	163.70
	*C*_max_ (ng/mL)	25.1	23.3	107.79	93.88	123.75
M2	AUC_0–∞_ (h·ng/mL)	453	498	90.94	69.32	119.31
	AUC_0–_*_t_* (h·ng/mL)	407	469	86.70	66.42	113.18
	*C*_max_ (ng/mL)	56.6	68.7	82.34	63.03	107.57
M7	AUC_0–∞_ (h·ng/mL)	7530	8340	90.26	73.82	110.36
	AUC_0–_*_t_* (h·ng/mL)	7410	8180	90.69	74.21	110.81
	*C*_max_ (ng/mL)	862	1150	75.12	60.17	93.79

#### 2.3.2. MAD study

**Figure 2.**
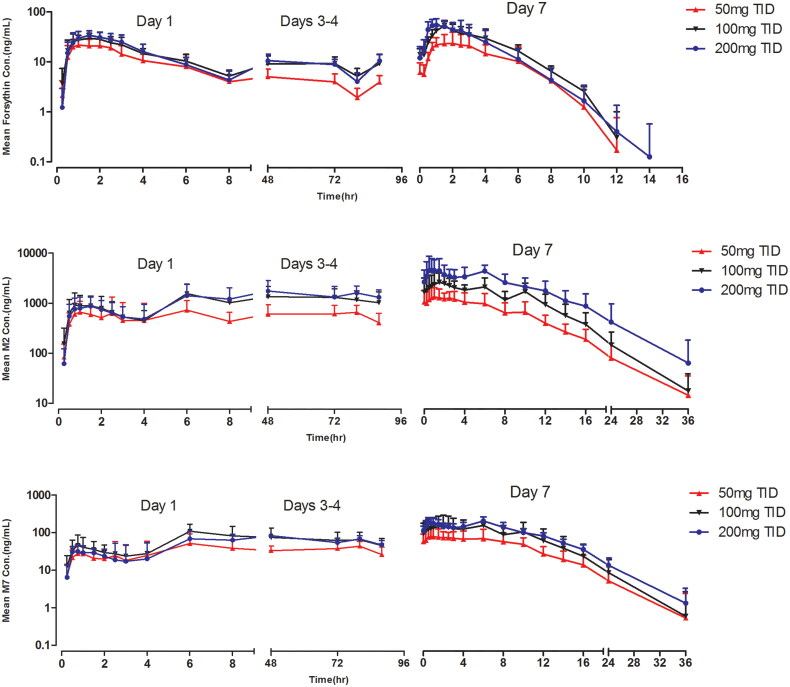
Pharmacokinetic profiles of forsythin, M2 and M7 in healthy subjects in multiple-ascending-dose study.

The mean forsythin and metabolite concentration–time profiles after multiple doses are presented in Figure 2. The plasma PK parameters for forsythin and its metabolites on days 1 and 5 are shown in Table 4. *R*_acc_ values were 1, 3 and 2 for forsythin, M2 and M7, respectively, indicating apparent plasma accumulation of M2 and M7 on day 5. Steady-state conditions were reached on day 3. The mean forsythin *C*_max_ and AUC_ss_ at steady state increased with dose (power model exponent *β*, 0.57 and 0.42, respectively). The exponents *β* for M2 and M7 were 0.81–0.84 and 0.55–0.59, respectively.

**Table 4.** Plasma pharmacokinetic parameters in the multiple-ascending-dose study.

PK parameters	Forsythin			M2			M7		
	50 mg TID	100 mg TID	200 mg TID	50 mg TID	100 mg TID	200 mg TID	50 mg TID	100 mg TID	200 mg TID
	(*N* = 9)	(*N* = 9)	(*N* = 9)	(*N* = 9)	(*N* = 9)	(*N* = 9)	(*N* = 9)	(*N* = 9)	(*N* = 9)
*T*_max(D5)_ (h) Mean(range)	1.50 (0.750-3.00)	1.50 (1.00-3.00)	1.50 (0.500-2.50)	2.50 (1.00-6.00)	2.00 (0.500-6.00)	3.00 (0.500-6.00)	3.00 (0.500-6.00)	2.00 (0.250-6.00)	2.50 (0.500-6.00)
*C*_max(D5)_ (ng/mL)	18.4 ± 7.35 (39.9)	32.8 ± 5.88 (17.9)	38.9 ± 7.75 (19.9)	1180 ± 307 (26)	2080 ± 821 (39.6)	3900 ± 2150 (55.1)	63.6 ± 31.7 (49.8)	110 ± 71.3 (64.6)	132 ± 49.9 (37.7)
*T*_1/2(D5)_ (h)	1.83 ± 0.326 (17.8)	1.67 ± 0.126 (7.6)	1.85 ± 0.459 (24.8)	5.18 ± 1.46 (28.1)	4.97 ± 1.53 (30.8)	4.94 ± 2.22 (45.1)	4.72 ± 2.28 (48.4)	4.98 ± 2.23 (44.7)	4.25 ± 2 (47)
AUC_0–t(D1)_ (h ng/mL)	93.6 ± 40.9 (43.7)	127 ± 31.0 (24.4)	132 ± 35.9 (27.1)	4290 ± 1980 (46.2)	7120 ± 2960 (41.6)	6910 ± 4020 (58.2)	249 ± 143 (57.6)	441 ± 240 (54.4)	306 ± 187 (61.0)
AUC_SS_ (h ng/mL)	74.1 ± 39.5 (53.4)	131 ± 37.1 (28.3)	129 ± 48.0 (37.3)	5730 ± 1780 (31.0)	10300 ± 3570 (34.7)	18400 ± 6950 (37.9)	321 ± 149 (46.2)	581 ± 374 (64.3)	711 ± 280 (39.4)
CL_ss_/F (L/h)	847 ± 438 (51.7)	806 ± 181 (22.4)	1860 ± 1040 (55.8)	9.7 ± 3.68 (37.9)	11.1 ± 4.75 (43)	12 ± 3.5 (29.1)	190 ± 90.4 (47.5)	226 ± 114 (50.5)	325 ± 133 (40.8)
*C*_min_ (ng/mL)	2.95 ± 1.91 (64.6)	4.54 ± 1.12 (24.7)	3 ± 1.49 (49.8)	327 ± 170 (52.1)	632 ± 396 (62.7)	1250 ± 639 (51.1)	19.8 ± 9.95 (50.3)	35 ± 35 (100)	52.4 ± 28.6 (54.6)
*C*_avg_ (ng/mL)	9.26 ± 4.94 (53.4)	16.4 ± 4.64 (28.3)	16.1 ± 6 (37.3)	717 ± 222 (31)	1290 ± 446 (34.7)	2290 ± 869 (37.9)	40.2 ± 18.6 (46.2)	72.7 ± 46.7 (64.3)	88.9 ± 35.0 (39.4)
*V*_z_/F(L)	2220 ± 1090 (48.9)	1950 ± 488 (25.1)	5450 ± 4840 (88.8)	67.5 ± 16.9 (25)	78.1 ± 30.7 (39.3)	84.4 ± 38.6 (45.7)	1150 ± 612 (53.4)	1540 ± 919 (59.6)	2030 ± 1320 (65.3)
*R*_ac_AUC_	0.823 ± 0.324 (39.3)	1.09 ± 0.389 (35.6)	0.975 ± 0.283 (29)	1.53 ± 0.605 (39.4)	1.75 ± 1.14 (65.2)	3.54 ± 2.16 (60.9)	1.6 ± 0.799 (50.0)	1.73 ± 1.48 (85.6)	2.98 ± 1.82 (61.1)
*R*_ac___Cmax_	0.752 ± 0.286 (38)	0.926 ± 0.249 (26.8)	1.04 ± 0.188 (18)	1.24 ± 0.563 (45.3)	1.48 ± 1.26 (84.9)	3.15 ± 1.8 (57.1)	1.24 ± 0.706 (56.9)	1.44 ± 1.79 (124.6)	2.04 ± 1.12 (54.8)
DF	180 ± 40.5 (22.5)	177 ± 31.9 (18)	247 ± 75.9 (30.7)	125 ± 33.3 (26.6)	115 ± 34.6 (30)	110 ± 23.1 (21.1)	109 ± 26.5 (24.3)	109 ± 41.5 (37.9)	92.8 ± 13 (14)

*N* = number of subjects analyzed; data are expressed as mean ± SD (CV%) unless otherwise specified.

## 3. Discussion

This Phase I trial assessed the safety, tolerability and PK characteristics of forsythin in healthy Chinese individuals. These findings indicate that single or multiple doses of forsythin (≤800 mg/day) are well tolerated in these individuals. No serious AEs or deaths were observed, and no AEs led to discontinuation of the study. The incidence or severity of all AEs was not correlated with the dose. The rates of AEs of forsythin were slightly lower than those of placebo in both the SAD and MAD studies. Forsythin did not show any safety risk compared to other drugs used to treat the common cold or influenza [15–17].

The PK results of the SAD study confirmed that forsythin is rapidly absorbed. It has a short half-life for TID. Increasing the forsythin dose in subjects resulted in less than dose-proportional increases in exposures, indicative of exposure saturation. After a single dose, forsythin showed moderate variability in AUC_0–∞_ and *C*_max_.

As concentrations for M1 were quite low due to the fast conjugation reaction, M1 was only analyzed in the 100 and 800 SAD cohorts. As trough concentrations are closely related to antipyretic function due to its mechanism, a doubling dose could be applied on the first dose to reach a steady state faster. Therefore, a doubling dose was applied in the 50 mg, and 100 mg MAD cohorts. Concentrations for forsythin 8 h post single dose were low (*T*_1/2_:1.79 ± 0.431) so that AUC_0-_*_t_* in the SAD cohort and AUC_0-8h(D1)_ in the MAD cohort could be comparable. However, AUC_0–_*_t_* for 50 mg SAD and AUC_0–8h(D1)_ for the MAD cohort were similar (95.6 ± 44.0 and 93.6 ± 40.9). Therefore, doubling the dose barely affected the PK characteristics of forsythin, indicating that doubling the dose would not help strengthen efficacy.

Steady-state for forsythin and metabolites was considered to have been reached by day 3 in the MAD study, which was consistent with *T*_1/2_. TID was chosen to elevate exposure and *C*_min_, and we found that *C*_8h_ decreased significantly in the SAD study. Systemic exposure to forsythin was similar on day 5 compared with day 1, with no significant accumulation over the 5-day doses, indicating that its PK properties are not time-dependent. Increasing the forsythin dose in subjects resulted in less than a dose-proportional increase in exposure to forsythin and M7. Exposure to M2 macrophages increased in a dose-dependent manner. Steady-state exposures to forsythin for 100 and 200 mg were similar, as the CLss/F of the 200 mg cohort was approximately twofold higher than that of the 100 mg cohort.

In the food effect study, the absorption of forsythin was delayed and the *T*_1/2_ values were similar. The AUC for forsythin in the fed state increased by approximately 40% compared to the fast state, while the AUC for M2 and M7 decreased by approximately 10%, indicating that forsythin could be taken after meals or not. It has been reported that drug absorption can be affected by food due to various changes in gastrointestinal physiology, such as gastric emptying time, pH and bile salt concentration [18]. The absorption of forsythin was improved by delayed gastric emptying, which may result from its low solubility and permeability.

Sulfotransferase (SULT) was previously reported to be the most active hepatic enzyme involved in the formation of M2. Compounds in the diet may inhibit the activities of SULT, thus decreasing the sulphation of forsythin [19].

Further clinical trials are required to determine the efficacy of forsythin in patients. A phase 2 study of forsythin in patients infected with influenza is currently underway. Exposures of forsythin in 100–200 mg TID cohorts are higher than these for efficacy dose cohorts in animal models. Therefore, considering safety and efficacy, forsythin 200 mg TID for 5 days was chosen in the phase 2 study.

Forsythin is safe and tolerable in healthy Chinese subjects. Forsythin is well-absorbed after single or multiple doses and is extensively metabolized. The sulphate conjugate M2 was the primary metabolite in the plasma. The preclinical pharmacodynamics, safety profile and PK characteristics of forsythin support further evaluations of its safety and efficacy in patients. Based on the data described above, 200 mg TID for five days was used for further clinical development.

## 4. Subjects and methods

### 4.1. Healthy subjects

The study was conducted at a single centre. Healthy Chinese individuals aged 18–45 years old, with body mass indexes of 18–28 kg/m^2^, were enrolled in this study. The eligibility of the participants was determined after a review of the following test results: laboratory tests –physical examination or ECG. Volunteers were excluded for clinically significant abnormal findings or a history of alcohol, smoking or drug abuse. All subjects were required to practice birth control and had no plans to conceive during the next six months.

The study was approved by the Research Ethics Board of the First Hospital of Jilin University, Jilin, China. This phase 1a study was performed at the Phase I Clinical Trial Unit of the First Hospital of Jilin University, Changchun, Jilin, China. This study was conducted between 21, June 2017 and 14, July 2018 in accordance with the Declaration of Helsinki and followed the principles of Good Clinical Practice. Written informed consent was obtained from all recruited volunteers. The clinical trial registration number was ChiCTR-IIR-17011570.

### 4.2. Study design and drug administration

This double-blinded, randomized, placebo-controlled phase 1a trial was comprised of three parts.

The SAD study was conducted in six sequential dosing cohorts (50, 100, 200, 400, 600 or 800 mg), with a total of 62 individuals. As no PK results have been previously reported, two subjects were enrolled and administered 50 mg. Sixty subjects in the SAD study were randomly assigned to receive forsythin or placebo, with an allocation ratio of 4:1 in each dose cohort. Randomization lists for the study were created using the SAS V9.4 software (SAS Institute, Cary, NC, USA). The participants were dosed in a fasted state. Tolerance was assessed on day 4 based on a medical review of the safety results. The next dose level was administered after reviewing the safety data.

In addition, 14 subjects from cohort A (*n* = 7) and cohort B (*n* = 7) were dosed with 100 mg to assess the food effect of forsythin in a two-period crossover food effect study with a 4-day washout. Subjects in cohort A were dosed 30 min after a high-fat meal in the first period and in a fasted state in the other period. The opposite was true for the subjects in cohort B.

A MAD study (50, 100 and 200 mg; *n* = 12 per dose cohort; TID for the first 4 days and a single dose for the 5th day) was conducted. For the 50 and 100 mg cohorts, the doubling dose was applied at the first dose to elevate trough concentrations. Subjects were dosed 30 min after a moderate-fat meal and randomly assigned to receive forsythin or placebo at an allocation ratio of 3:1. The sponsor, Dalian Fusheng Institute of Natural Medicine, provided forsythin and placebo in identical packaging and appearance to ensure that the participants and investigators were blinded to the group allocation and that the doses were chosen according to the PK and safety results of the SAD study.

### 4.3. Blood, urine and faeces sampling and drug analysis

In SAD study, blood samples were collected in chilled collection tubes containing the anticoagulant K_2_-ethylenediaminetetraacetic acid (EDTA) at the baseline (within 30 min prior to dosing) and 0.25, 0.5, 0.75, 1, 1.5, 2, 3, 4, 6, 8, 10, 12, 14, 16, 24 and 36 h after dosing. For the food effect study, the samples were drawn at the same time points on days 1 and 4. In the MAD study, blood samples were drawn from 30 min before dosing to 8 h after dosing on day 1 and from 30 min before dosing to 36h after dosing on day 5. The trough concentration samples were collected pre-dose on day 3 after the first administration and on day 4 after all administrations. Plasma samples were then prepared. All samples were maintained at −70 °C until analysis.

In a previous study, forsythin underwent extensive metabolism *via* hydrolysis and further sulphation after a single dose. The detailed deduction process of the metabolites M1, M2 and M7 were reported [14]. Three metabolites were confirmed by comparison with reference substances: aglycone M1, M1 sulphate (M2) and M1 glucuronide (M7). A validated liquid chromatography–tandem mass spectrometry (LC–MS/MS) method was used to quantify the concentrations of forsythin and its main metabolites, M1, M2 and M7, in human plasma, urine and faecal samples. The LC–MS–MS data, and structures of the metabolites M1, M2 and M7 were also reported [14].

### 4.4. PK analysis

The PK parameters were calculated with WinNonlin 7.0 (Certara, Princeton, NJ, USA) using noncompartmental analysis. As the PK parameters of the SAD study were previously reported, calculations of PK parameters for the MAD study and the food effect study were conducted in this report. In the MAD study, PK parameters of forsythin and its main metabolites M2 and M7, including *C*_max_, *T*_max_, AUC_0–_*_t_*, AUC_0–∞_, *T*_1/2_, CL/*F*, *V*_Z_/*F*, *C*_min,ss_, *C*_avg,ss_, AUC_ss_, drug fluctuation coefficient (DF) and accumulation ratio (*R*_acc_) were estimated. The *R*_acc_ at steady-state after multiple doses was calculated as follows: (AUC_0–8_ or *C*_max_ on day 5)/(AUC_0–8_ or *C*_max_ on day 1). Steady-state status was determined by visual inspection of trough concentrations (*Cτ*) on days 3, 4 and 5.

### 4.5. Evaluation of tolerability and safety

Safety and tolerability were assessed in all subjects who received at least one dose of forsythin. Safety was evaluated based on the presence of AEs, clinical laboratory tests (hematology, urinalysis and blood chemistry), vital signs, physical examinations and 12-lead electrocardiography.

### 4.6. Statistical analysis

Statistical analyses were performed using the SAS software (version 9.4; SAS Institute, Inc., Cary, NC, USA). Descriptive statistics were applied to the demographic parameters and safety data. Formal comparisons between the dose cohorts were not performed in this study. Forsythin and metabolite plasma concentrations, along with PK parameters, were summarized and compared among the different dose cohorts using descriptive statistics. To examine dose proportionality for *C*_max_ and AUC, a power model was fitted to the data to examine dose proportionality for *C*_max_ and AUC [20].

In the food effect study, *C*_max_ and AUC_0–∞_ were compared between the fasted and fed states using a mixed-effects model for log-transformed PK values with a treatment sequence, period, and treatment as fixed effects, and subjects nested with a sequence fitted as a random effect. The 90% CI of the geometric mean ratios (GMR) for the variables *C*_max_ and AUC_0–∞_ were calculated.

This study was registered at https://www.chictr.org.cn under the identifier ChiCTR-IIR-17011570.

## Data Availability

The authors confirm that data supporting the findings of this study are available within the article.
